# Effects of a novel foot–ankle orthosis in the non-operative treatment of unicompartmental knee osteoarthritis

**DOI:** 10.1007/s00402-016-2500-2

**Published:** 2016-07-08

**Authors:** Björn Menger, Andreas Kannenberg, Wolf Petersen, Thore Zantop, Ingo Rembitzki, Hartmut Stinus

**Affiliations:** 1 Department of Orthopaedic and Trauma Surgery, School of Medicine, University of Göttingen, Göttingen, Germany; 2 Otto Bock Health Care, Duderstadt, Germany; 3 Klinik für Orthopädie und Unfallchirurgie, Martin Luther Krankenhaus, Berlin, Germany; 4 Sporthopaedicum Straubing, Straubing, Germany; 5 Deutsche Sporthochschule Köln, Cologne, Germany; 6 Orthopaedicum Northeim, Northeim, Germany

**Keywords:** Osteoarthritis, Unloader brace, Non-operative treatment, Varus knee, Malalignment, Knee adduction moment

## Abstract

**Introduction:**

Unloader braces are non-surgical treatment options for patients with unicompartmental knee osteoarthritis (OA). However, many patients do not adhere to brace treatment because of complications related to discomfort and poor fit. An alternative to knee bracing is an ankle–foot orthosis (AFO) with a lever arm that presses the lower leg into valgus or varus. The aim of this study is to evaluate the clinical benefits of this AFO for patients with unicompartmental knee OA.

**Materials and methods:**

Twenty-three patients with knee OA were enrolled in this observational study. The primary clinical outcome measure was the Western Ontario and McMasters Universities Arthritis Index (WOMAC) total score. Secondary outcome measures included WOMAC subscores, visual analogue pain scale, activity restriction and complication rate. Clinical scores were collected at start and 3, 6, 9, and 12 months after enrollment. Statistical evaluation was performed using the Student’s *t* test.

**Results:**

Of the patients enrolled, 83 % suffered from medial compartment OA. Most patients had Grade II OA according to the Kellgren and Lawrence classification. WOMAC total score, both subscores and visual analogue pain scale were significantly improved over time. Patients also noted a reduction in restrictions to activities of daily living and sport-related activities while using the AFO. No patients discontinued orthosis use because of adverse effects. Two types of complications were noted: discomfort or light pressure sores around the ankle (7 patients), and wear and tear of the shoe in which the AFO was worn (14 patients).

**Conclusions:**

This observational study suggests that this AFO is effective at significantly reducing pain and stiffness as well as improving the physical function of patients with mild to moderate unicompartmental osteoarthritis of the knee.

## Introduction

Osteoarthritis (OA) is the most common joint disease, with a prevalence of 6 % [7]. OA prevalence increases with age [1].

In patients with knee OA, the medial joint compartment is more commonly affected than the lateral compartment [7]. During normal gait, except for a brief abduction moment after initial heel contact, the knee joint is subjected to an external adduction moment throughout the stance phase [4, 5, 8–12, 16, 19, 20]. This adduction moment is responsible for the load shift from the lateral to medial compartment. This load shift even occurs in the presence of a valgus knee deformity [3–5, 11, 13, 20–22]. The combination of adduction moment and increased medial compartment loads are thought to be responsible, in part, for the high incidence of medial knee OA.

The amplitude of the external adduction moment applied to the knee depends on the joint’s mechanical alignment and ground reaction forces. In patients with medial knee OA, the medial joint space narrows as a result of cartilage degeneration, which shifts the mechanical alignment of the knee into varus. This shift can result in an even greater external adduction moment [22], unless the patient develops a compensatory gait pattern that involves toeing out. A reduction in proprioception [2, 19] that occurs regularly in patients with OA predisposes the joint to abnormal kinematics. These changes are compounded by an increased external varus moment that shifts more load onto the affected compartment, which may also promote degeneration.

A permanent solution for the correction of lower extremity malalignment is a high tibial osteotomy [17]. However, surgical treatment is always associated with risks such as thrombosis, embolism and infection. Conservative treatment options include the use of lateral wedged insoles [2, 9, 14, 20] and knee unloader braces [5–7, 10–12, 16, 22], which can be adjusted to produce a valgus thrust to unload the medial compartment or a varus thrust to unload the lateral compartment. A systematic review of biomechanical studies concluded that knee unloader braces reduce external moments acting on the knee joint [16].

Randomized controlled trials have shown that knee bracing results in improved knee function compared with no bracing in patients with OA and varus malalignment [10]. However, a side effect of brace treatment is skin irritation caused by the condylar pads or straps, which may lead to treatment non-adherence [22].

**Fig. 1:**
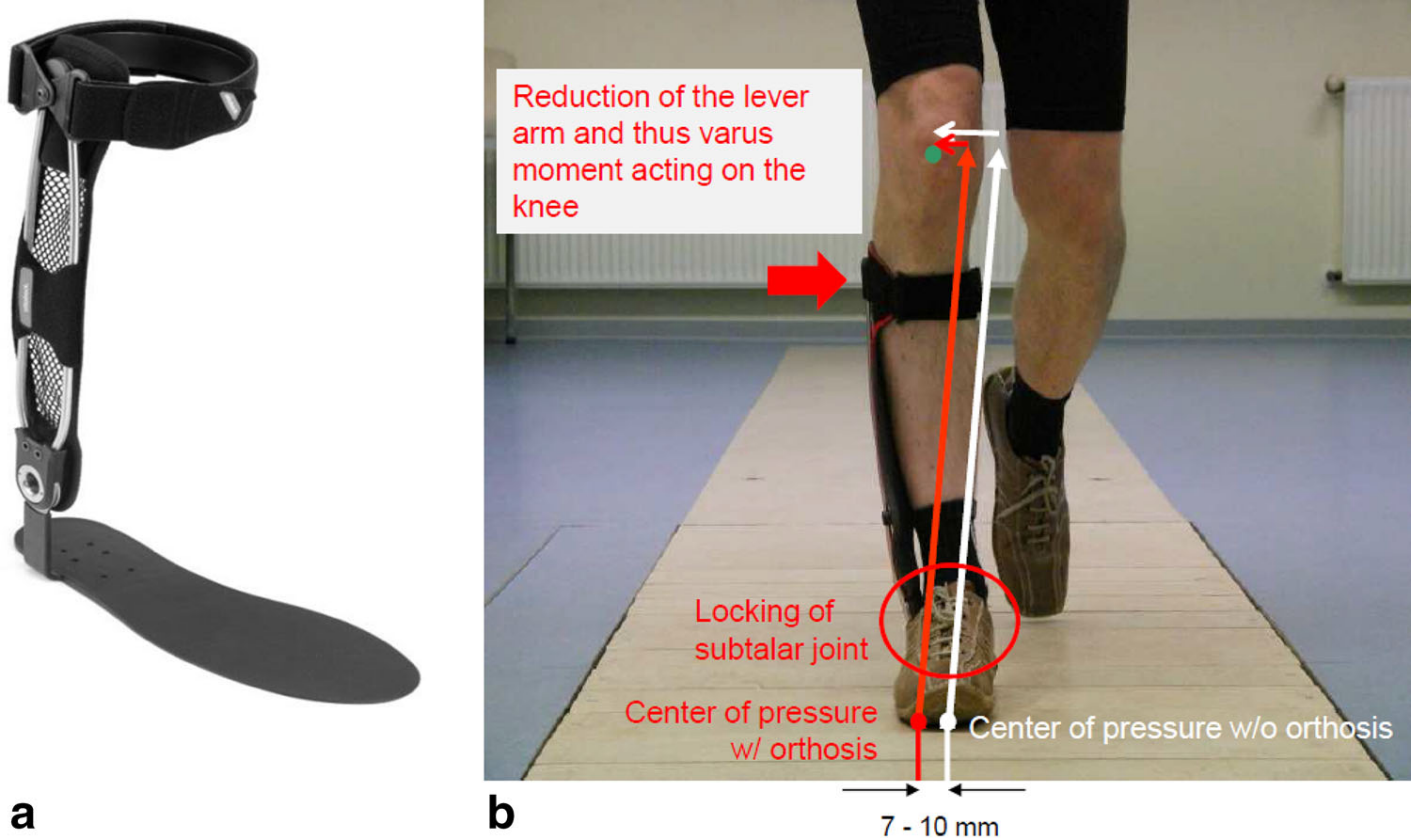
Mechanisms and effects of a novel ankle–foot orthosis (Agilium Freestep^®^, Otto Bock, Duderstadt, Germany) in the management of medial knee osteoarthritis

A new strategy for correcting varus malalignment is to apply an external valgus force to the knee with an ankle-foot orthosis (AFO) (Fig. 1). This AFO (Agilium Freestep®, Otto Bock, Duderstadt, Germany) consists of a non-flexible insole that is connected to a lever with a pad, which in turn applies a valgus force to the lower leg [21, 22]. Skin irritation should be minimized because of the lack of condylar pads.

Recent biomechanical studies have shown that this new brace concept is effective at reducing the knee abduction moment compared with treatment with insoles alone [6, 21]. However, there are no clinical studies that examine the effects of this new brace.

Here we assessed if this AFO has beneficial effects on the symptoms of patients with unicompartmental knee OA. Our hypothesis was that the use of an AFO improves OA related symptoms as measured with the Western Ontario and McMasters Universities Arthritis Index (WOMAC) in patients with unicompartmental knee OA.

## Methods

### Study design

This prospective cohort study was conducted at a private practice, the Orthopaedic Department of the Martin Luther Hospital, and the Department of Orthopedics and Trauma Surgery at the University of Göttingen.

A sample size calculation revealed that the inclusion of 22 patients would permit statistically significant improvements in WOMAC score in this study. Calculating a potential dropout rate of three patients, 25 patients were included in this study. Inclusion criteria were: (1) age over 18; and (2) unicompartmental knee OA (medial or lateral). Medial OA was defined as pain located in the medial joint space in combination with radiographic signs of OA (Grade I or higher). Radiographic signs of OA were assessed with standing X-rays using the Kellgren and Lawrence classification (Grades 0–IV). Exclusion criteria included the following: (1) no knowledge of the German language; and (2) pain that was not caused by medial or lateral knee OA. Patients who qualified as study participants based on these inclusion and exclusion criteria were informed about the study. This discussion included general information about medial OA and a description of the study protocol.

All patients included in this study gave their written informed consent to participate and to have their outcomes documented using standardized questionnaires. This study design was approved by the medical ethics committee of the medical faculty of the Charité—Universitätsmedizin Berlin (EA 1/069/15, 26.3.2015).

After enrollment, each patient had an ankle–foot orthosis fitted by a technician. Each patient´s demographic data (name, sex, and age), baseline medical findings (radiographic assessment of OA, leg alignment and treatment history) and primary and secondary outcome measures were collected.

### The ankle–foot orthosis (AFO)

The AFO (Agilium Freestep, Otto Bock HealthCare) is a CE-certified medical device approved in the European Union for the treatment of medial and lateral unicompartmental knee osteoarthritis (OA). The orthosis is available in four sizes each for the left and right leg, and is individually adjusted by a technician. The size was selected based on the shoe size of the patient. This AFO shifts the center of pressure (CoP), defined as the application point of the ground reaction force (GRF) under the foot, 7–10 mm lateral (Fig. 1) or medial in cases of lateral knee compartment OA. It also locks the subtalar joint and keeps the calf upright to ensure proper force transmission to the knee joint. As a result, the lever arm of the GRF, and thus the external frontal moment acting on the knee, is reduced.

On the contralateral side, an insole was applied to balance the base plate of the orthosis.

Participants were instructed to wear the orthosis for as long as possible. The duration of therapy was not limited.

Additional conservative therapies were permitted. A surgical procedure during the follow-up period on the ipsilateral lower extremity was criteria for study exclusion.

### Outcome measures

Our primary outcome measure was the WOMAC total score. Our secondary outcome criteria were the WOMAC subscores, pain measured using a visual analogue scale (VAS), adverse effects and restrictions to activities of daily living (ADL) and sport-related activities. ADL and sport-related activity restriction were assessed using a four-item Lickert scale (none, little, moderate and severe). Other secondary outcome measures included the duration of average daily AFO use (in hours), occurrence of and reasons for longer-term (≥2 week) discontinuation of orthosis use, use and dosage of non-steroidal anti-inflammatory drugs (NSAIDs) and adverse events related to orthosis use (including discomfort, sore limbs and wear and tear).

Follow-up visits were scheduled after 3, 6, 9, and 12 months.

### Statistics

Most data were evaluated with descriptive statistics. WOMAC total and subscores, as well as knee pain measured with VAS underwent comparative statistics with the Student’s *t* test for paired samples using WinStat for Microsoft Excel (Redmond, WA, USA).

## Results

### General demographic and baseline health data

Twenty-five patients were enrolled in this study. Two patients dropped out after 2 and 5 months. The first patient terminated participation because of bunion surgery, and the second patient moved to a different region of Germany. The complete datasets of 23 patients were available for final intention-to-treat analysis, which used a last-observation-carried-forward method to extrapolate the data points for the two drop-outs.

Out of the 25 enrolled patients, 15 (60 %) were male and 10 (40 %) were female, with a mean age of 60.5 ± 11.7 years. Fifteen patients (60 %) had OA of the left knee and 10 (40 %) had an affected right knee.

The medial compartment was affected by osteoarthritis in 21 patients (83 %), whereas the lateral compartment was affected in four patients (17 %). Based on the Kellgren and Lawrence classification, at baseline eight patients (33 %) had Grade I osteoarthritis, 14 (55 %) had Grade II osteoarthritis and three (12 %) had Grade III osteoarthritis.

Ten patients (40 %) demonstrated a neutral leg axis, 11 (44 %) had a varus deviation and four (12 %) had a valgus deviation.

### Primary outcome measure

**Fig. 2:**
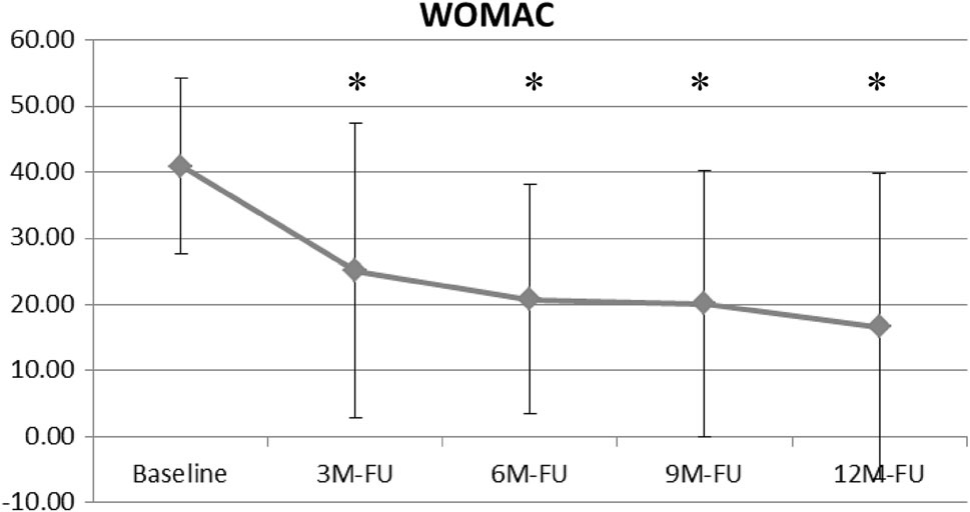
Change in WOMAC total score from baseline to 12-month follow-up with AFO use (* = statistically significant difference compared with baseline)

As presented in Fig. 2, the WOMAC total score was significantly improved following AFO use at all follow-up visits compared with baseline scores (40.9 ± 13.4). After 3 months the intervention group had a WOMAC of 25.1 ± 22.3 (*p* < .03), followed by 20.8 ± 17.3 (*p* < .0001) at the 6-month follow-up. It continued to trend down at the 9- (20.2 ± 20.1, *p* < .0001) and 12-month follow-ups (16.6 ± 23.3, *p* < .001).

### WOMAC subscores

**Fig. 3:**
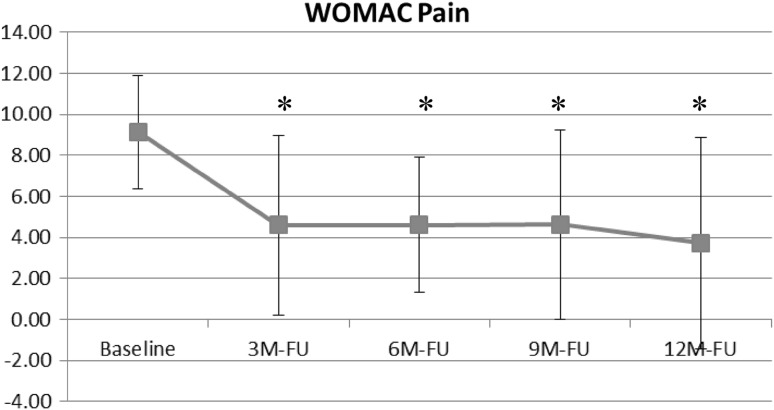
Change in WOMAC pain subscore from baseline to 12-month follow-up with AFO use (* = statistically significant difference compared with baseline)

The WOMAC pain subscore was significantly improved during AFO use (Fig. 3). A baseline of 9.1 ± 2.8 improved to 3.7 ± 5.1 (*p* < .004) at the 12-month follow-up.

**Fig. 4:**
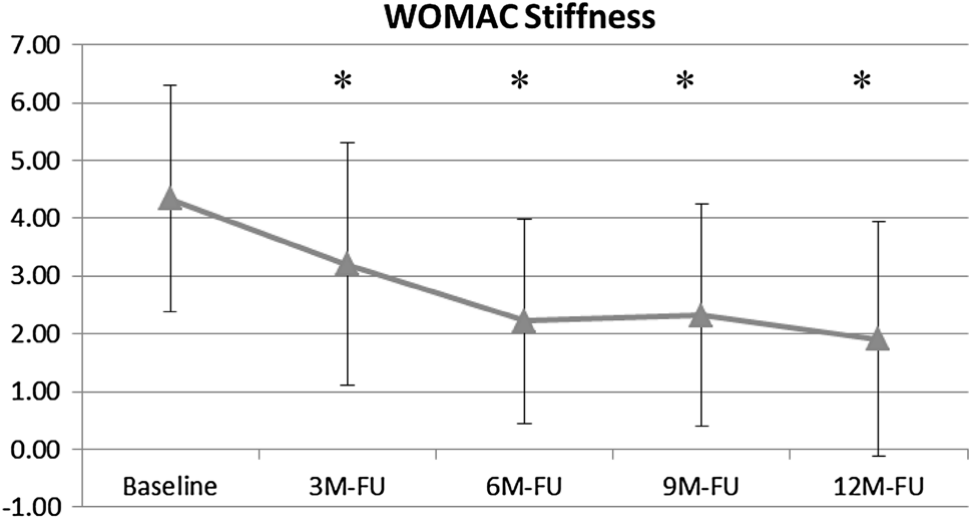
Change in WOMAC stiffness subscore from baseline to 12-month follow-up with AFO use (* = statistically significant difference compared with baseline)

The WOMAC stiffness subscore is shown in Fig. 4. This subscore also improved significantly from baseline (4.3 ± 2.0) to the 6-month (2.2 ± 1.8; *p* < .0004), 9-month (2.3 ± 1.9; *p* < .0003), and 12-month (1.9 ± 2.0; *p* < .0003) follow-ups.

**Fig. 5:**
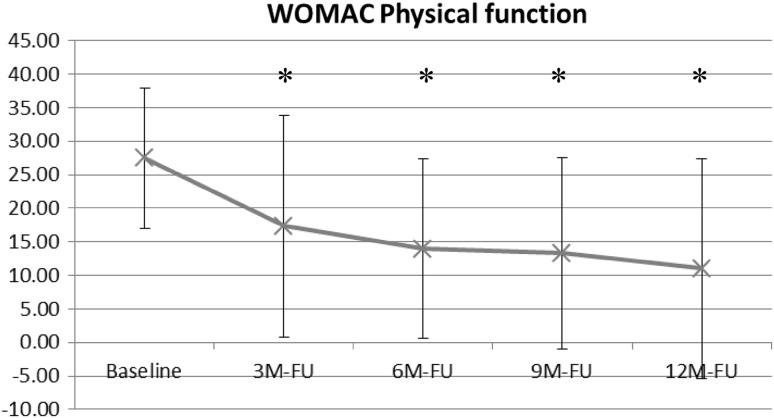
Change in WOMAC physical function subscore from baseline to 12-month follow-up with AFO use (* = statistically significant difference compared with baseline)

Figure 5 demonstrates the WOMAC physical function subscore. This score significantly decreased from baseline (27.4 ± 10.5) to the 6-month follow-up (13.9 ± 13.3, *p* < .0003), the 9-month follow up (13.2 ± 14.2, *p* < .0002) and the 12-month follow-up (11.0 ± 16.3, *p* < .0002).

### Knee pain (VAS)

**Fig. 6:**
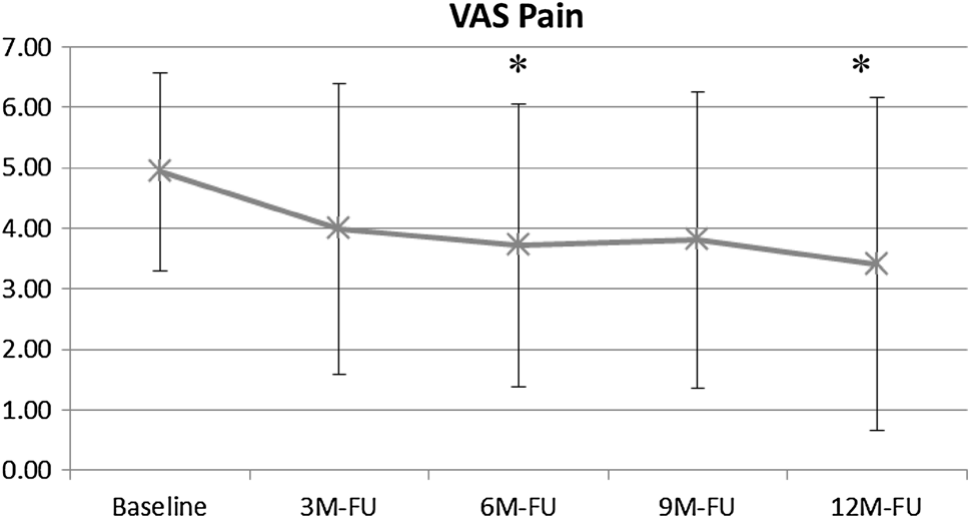
Change in VAS pain score from baseline to 12-month follow-up with AFO use (* = statistically significant difference compared with baseline)

Quantitative VAS pain assessment (Fig. 6) was significantly improved at the 6-month (3.7 ± 2.3, *p* < .03) and 12-month follow-ups (3.4 ± 2.8, *p* < .04) compared with baseline (4.9 ± 1.6).

### NSAID use

Fourteen patients used NSAIDs prior to the start of the study, 10 of whom reported a dosage reduction of 50 % or more when using the orthosis.

### ADL and sport-related activity restrictions

**Fig. 7:**
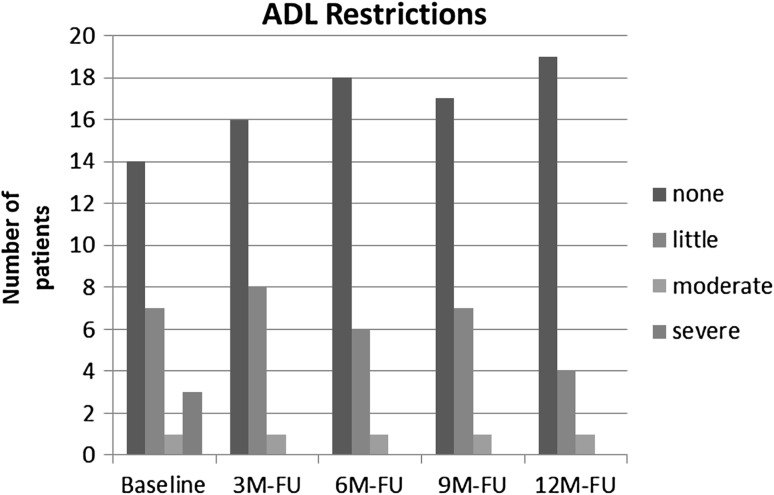
ADL restriction over time with AFO use as measured with a four-point Lickert scale

**Fig. 8:**
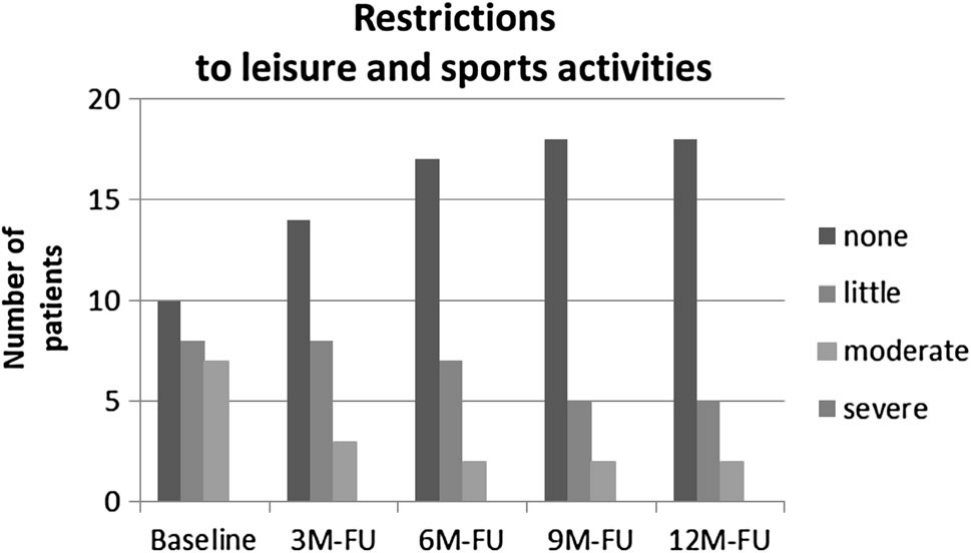
Sport and leisure activity over time with AFO use as measured with a four-point Lickert scale

Perceived restrictions to activities of daily living and sport-related activities decreased over time during AFO use (Figs. 7, 8).

### Orthosis use

Seventeen patients wore the AFO for 4–8 h daily. One patient wore it less than 4 h a day and five patients extended their usage to more than 8 h daily.

No patients discontinued their use of the orthosis completely. Seven of the 23 patients reported non-use of the orthosis for 14 days during the 12-month follow-up period. Two patients had to interrupt their orthosis use during hospital stays unrelated to their knee OA. Four patients did not use their orthosis during a vacation, and one patient had to interrupt AFO use because of a technical defect that required a repair.

### Adverse events

During the 12-month follow-up period, two types of adverse events were observed. Discomfort or light pressure sores around the ankle were seen in seven patients. These adverse effects were observed within the first 4 weeks after initial orthosis fitting. All complaints were well managed with additional adjustments or orthosis padding by a technician. Wear and tear of the shoe on the AFO side was seen in 14 patients after longer AFO use, usually at the 9- and 12-month follow-up visits.

## Discussion

This is the first clinical outcome study of the use of a new AFO for unicompartmental knee OA. The results of this study confirmed our hypothesis. The AFO improved WOMAC total score as well as its three subscores: pain, stiffness and physical function.

Improvements in WOMAC total and subscores were about 50 % after 6 and 12 months, which was roughly the efficacy of studies that evaluated knee unloader braces [3, 5, 7].

The improvement in pain VAS score of about 30 % after AFO use, which was statistically significant at the 6- and 12-month follow-ups, was also comparable to that observed in clinical trials of knee unloader braces [6, 10, 12, 18].

These clinical effects confirm previous biomechanical studies that have shown that an AFO has the potential to reduce the knee adduction moment in patients with medial OA [6, 20]. Fantini et al. [6] found significant decreases in knee adduction moment, knee lever arm and joint alignment in the frontal plane with an AFO. Schmalz et al. [20] found that an AFO that is rigid in the frontal plane significantly reduced the maximal adduction moment of the knee from 0.54 Nm/kg to 0.38 Nm/kg.

The clinical and biomechanical effects of clinical interventions such as unloader braces, which are recommended by the Osteoarthritis Research Society International guidelines for the treatment of knee OA, are well documented [3, 9]. A downside of unloader brace treatment is the high rate of therapy discontinuation. In one prospective randomized trial, the compliance with brace treatment was only 45 % [23]. Causes for the discontinuation of brace treatment include a lack of therapeutic benefit or adverse effects such as abrasions, bruises, sores and blisters at the level of the condylar knee pads [23]. In our study, seven of 23 patients reported a partial discontinuation of AFO use for 14 days or longer during the course of their study participation. In six of these patients the cause for discontinuation was not related to the orthosis (vacation or unrelated hospital stay). However, discomfort or light pressure sores around the ankle were evident in seven patients within the first 4 weeks of AFO use. These problems were managed with additional adjustments to the orthosis by a technician. The second type of adverse events, wear and tear of the shoe in which the AFO was worn, was seen in 14 patients after longer AFO use, typically at the 9- and 12-month follow-up visits.

The optimal daily brace use time has not yet been determined [23]. Van Raaij et al. [23] defined compliance as brace use of 6 h a day. In our study the majority of patients (17/23) wore their orthosis for 4–8 h per day [23].

Our results, in combination with previous biomechanical findings [6], suggest that this new AFO design is a sound alternative to conventional knee unloader braces. However, this study has two important limitations. First, this study is not a prospective randomized trial. We lacked a control or placebo group. Prospective randomized trials are needed to further confirm the benefits of this AFO. However, our study design allowed us to avoid several disadvantages of randomized controlled trials. Lack of generalizability is a major problem for randomized clinical trials (RCTs) [15]. Normally, only a small percentage of the patients can typically be enrolled in an RCT because of strict enrollment criteria [15, 18]. The participation rate of some RCTs is less than 15 % [15]. RCT enrollment can therefore be far from the “real world”, making findings difficult to translate into the general population. In our study the eligibility criteria were not highly selective, and patients who were recruited were representative of the patients in our local community. The second limitation of our study is that the examiner was also the caregiver, and the one who informed the patient about the aims of the study. Our study would have been stronger if the assessor was blinded during outcome measurement.

In conclusion, the results of our study suggest that the AFO evaluated here is effective at significantly reducing the pain and stiffness and improving the physical function of patients with mild to moderate unicompartmental osteoarthritis of the knee. The clinical benefits of this AFO appear to be similar to those demonstrated in clinical trials of knee unloader braces, although our study suggests that overall patient compliance with AFOs may be superior to unloader braces. However, additional prospective randomized studies are needed to further characterize this AFO in the treatment of unicompartmental OA.
